# A Case of a Recurrent Subperiosteal Orbital Abscess Secondary to Pansinusitis

**DOI:** 10.51894/001c.144418

**Published:** 2025-09-30

**Authors:** Ruth Antony

**Affiliations:** 1 Department of Ophthalmology Henry Ford Warren

## 63

### INTRODUCTION

Extensive sinus disease is known to be causative for subperiosteal orbital abscesses. In many cases, intravenous antibiotic therapy alone is insufficient, and subsequent surgical drainage is required. Herein we present a patient with recurrent orbital abscesses secondary to extensive pansinusitis.

### CASE DESCRIPTION

A 14-year-old male presented to the emergency department with a one-day history of headache and left-sided periorbital edema. He endorsed intermittent diplopia, and denied ocular pain or pain with extraocular movements. Initial exam revealed left-sided proptosis and EOM restriction in all gazes. Ophthalmic vitals including visual acuity, pupillary function, and intraocular pressure were normal. He was afebrile and initial labs revealed no abnormalities. Empiric antibiotic treatment was initiated. Computed tomography (CT) scan revealed a 11.93 cm3 subperiosteal abscess, with anterior and inferior displacement of the globe, and extensive frontal sinus disease. A combined functional endoscopic sinus surgery and orbital exploration with abscess drainage was conducted with samples collected for microbiology. Cultures grew Streptococcus anginosus and Eikenella corrodens, and antibiotic coverage was targeted. On postoperative day two, recurrent periorbital edema was noted. A repeat CT scan suggested reaccumulation of abscess fluid, and repeat orbitotomy was conducted along with penrose drain placement. The drain remained in place for three days before self-removal by the patient; oculoplastics subsequently closed the wound with sutures. Two-days later, CT revealed a recurrent superficial abscess measuring approximately 5.22 cm3. The wound was reopened to allow pus extrusion, and remained open for further draining. The patient’s hypoglobus began to resolve. Pain and diplopia improved in the following days. The patient was discharged with instruction to follow-up in the outpatient setting.

### CONCLUSIONS

Management of subperiosteal orbital abscesses secondary to sinus disease often requires a combined surgical approach consisting of sinus surgery and abscess drainage, in addition to systemic antibiotic therapy. It is recommended for a drain to be left in place for 24 to 48 hours. If meaningful resolution of the abscess is not noted in this time-frame, further surgical exploration and drainage may be indicated. Finally, if the abscess continues to recur, the wound may require being left open to facilitate prolonged drainage.

